# An integrated strategy for deciding open versus laparoscopic hepatectomy for resectable primary liver cancer

**DOI:** 10.1186/s12885-023-10630-x

**Published:** 2023-02-27

**Authors:** Yizhen Fu, Zhenyun Yang, Zili Hu, Zhoutian Yang, Jinbin Chen, Juncheng Wang, Zhongguo Zhou, Li Xu, Minshan Chen, Yaojun Zhang

**Affiliations:** 1 Sun Yat-Sen University Cancer Center; State Key Laboratory of Oncology in South China; Collaborative Innovation Center for Cancer Medicine, Guangzhou, Guangdong 510060 P. R. China; 2 Department of Liver Surgery, Sun Yat-Sen University Cancer Center, 651 Dongfeng Road East, Guangzhou, Guangdong 510060 P. R. China

**Keywords:** Hepatocellular carcinoma, Laparoscopic hepatectomy, Surgery, Nomogram, Risk

## Abstract

**Background:**

Laparoscopic liver resection (LLR) is now widely performed in treating primary liver cancer (PLC) and yields equal long-term and superior short-term outcomes to those of open liver resection (OLR). The optimal surgical approach for resectable PLC (rPLC) remains controversial. Herein, we aimed to develop a nomogram to determine the most appropriate resection approach for the individual patient.

**Methods:**

Patients with rPLC who underwent hepatectomy from January 2013 to December 2018 were reviewed. Prediction model for risky surgery during LLR was constructed.

**Results:**

A total of 900 patients in the LLR cohort and 423 patients in the OLR cohort were included. A history of previous antitumor treatment, tumor diameter, tumor location and resection extent were independently associated with risky surgery of LLR. The nomogram which was constructed based on these risk factors demonstrated good accuracy in predicting risky surgery with a C index of 0.83 in the development cohort and of 0.76 in the validation cohort. Patients were stratified into high-, medium- or low-risk levels for receiving LLR if the calculated score was more than 0.8, between 0.2 and 0.8 or less than 0.2, respectively. High-risk patients who underwent LLR had more blood loss (441 ml to 417 ml) and a longer surgery time (183 min to 150 min) than those who received OLR.

**Conclusions:**

Patients classified into the high-risk level for LLR instead undergo OLR to reduce surgical risks and complications and patients classified into the low-risk level undergo LLR to maximize the advantages of minimally invasive surgery.

**Trial registration:**

This study was registered in the Chinese Clinical Trial Registry (registration number: ChiCTR2100049446).

**Supplementary Information:**

The online version contains supplementary material available at 10.1186/s12885-023-10630-x.

## Background

Primary liver cancer (PLC) is the sixth most commonly diagnosed cancer and the fourth leading cause of cancer death worldwide as well as the second most lethal cancer in China [1, 2]. To date, surgical resection, liver transplantation and radiofrequency ablation remain curative treatments for PLC, especially hepatocellular carcinoma (HCC) [3, 4].

Liver resection (LR) is recommended for a single HCC of any size with preserved liver function and sufficient remnant liver volume [3]. Open liver resection (OLR) is the standard approach for HCC resection; however, the introduction of laparoscopic liver resection (LLR) brought a major change to surgical practice for liver cancer. In the first decade of the twenty-first century, LLR remained controversial because of its technical difficulty in terms of R_0_ radical resection, a sufficient resection margin and its oncological outcomes, namely, tumor seeding and disease-free survival [5, 6]. However, due to improvements in techniques and equipment, LLR is now widely performed for the treatment of HCC in most cancer centers and yields equal long-term outcomes to those of OLR. It has been reported that LLR is associated with less blood loss, fewer postoperative complications and fewer hospital stays with no compromise to recurrence and survival among patients with either HCC or colorectal liver metastasis, regardless of cirrhosis status [6–13]. As for intrahepatic cholangiocarcinoma (ICC), the second most common PLC, it has also been reported that LLR reduces intraoperative blood loss and postoperative hospital stay with no difference in postoperative morbidity and mortality within 30 days as well as long-term outcomes [14, 15].

However, the decision of the surgeons to perform LLR or OLR is based only on their experience or preference in the absence of randomized controlled trials (RCTs). In some selected patients, the laparoscopic approach could be the first choice. The Southampton Guidelines advocate that LLR should be considered standard practice for lesions in the left lateral segments [16]. By contrast, LLR must be performed with great caution when lesions are located in the “*Difficult Segments* (Couinaud Segment, Sg 1, 4a, 7 and 8)”, as the anatomical structures of those segments are highly complex and the whole procedure requires advanced expertise. Hence, it is particularly necessary to establish a strategy to decide which resection approach is favorable for both patient outcomes and surgical processes. Herein, we sought to develop an algorithm to distinguish high-, medium- and low-risk patients with resectablePLC (rPLC) who underwent LLR and determine the most appropriate resection approach for the individual patient.

## Methods

### Patients and data collection

From January 2013 to December 2018, patients who underwent hepatectomy and were pathologically diagnosed with PLC (including HCC, ICC and combined HCC-ICC) in our center were enrolled as candidates. According to the BCLC staging system, only BCLC stage 0 or A patients are recommended for liver resection [3]. However, an increasing number of studies have confirmed that patients with multiple nodules can benefit from surgical resection, even out of the Milan Criteria [17]. Thus, in this study, patients with 2 to 3 tumors and a maximum diameter larger than 3 cm were also included. Moreover, for patients with a large tumor burden, an adequate remnant liver volume might not be obtained, resulting in a higher incidence of postresection liver failure and a less sufficient resection margin [18, 19]. Hence, rPLC was finally defined as up to 3 tumors with a maximum diameter no larger than 10 cm, without macrovascular invasion or extrahepatic metastasis. Additional inclusion criteria were as follows: (1) a performance status (PS) score of 0 to 2; (2) preserved preoperative liver function (Child–Pugh Class A); (3) no other concurrent malignancies. The exclusion criteria were as follows: (1) combined liver ablation or surgery of other organs during the operation; (2) received LLR at first yet conversed to OLR in the surgery procedure; (3) a previous abdominal surgical history other than first hepatectomy, such as cholecystectomy.

Data on patient demographics, laboratory tests, operations, comorbidities and tumor pathology were prospectively collected in the medical records and retrospectively reviewed. This study was approved by the ethics committee of the Sun Yat-sen University Cancer Center (SYSUCC). Signed informed consent for the use of data for research purposes was obtained from patients before treatment.

### Surgical procedures

All LLRs and OLRs were performed by the same team led by Dr. Chen and Dr. Zhang with the experience of more than 100 successful LLRs and 1000 successful OLRs at the time of 2013. A standard operation procedure was utilized in both LLR and OLR.

In OLR, the patient was placed in the supine position and monitored under general anesthesia. A right subcostal incision or midline incision was adopted depending on the tumor location, and after separating ligaments around the liver, intraoperative ultrasonography was used to identify tumor boundaries and potential satellite nodules or vascular invasion. The liver parenchyma was cut using a harmonic scalpel collaborating with controlled low central venous pressure. Total hepatic inflow control was performed through the intermittent Pringle maneuver to reduce blood loss during transection. The cut surface of the liver and abdominal cavity was washed with a large amount of sterile water after careful hemostasis, and an abdominal drain was deployed when total blood loss was more than 200 ml or bile leakage was suspected.

In LLR, the patient lied in the Reverse Trendelenburg position and was raised by a pillow under the right side of the back when the tumor was located in the right lobe of the liver. One 12-mm trocar was placed through the periumbilical abdomen as the thoroughfare for laparoscopy and carbon dioxide to maintain the pneumoperitoneum. Additional two to four 5-mm or 12-mm trocars were placed as needed. The primary surgeon stood on the right side of the patient, and two assistants stood on the left side. Intraoperative ultrasonography was routinely used to guide the resection planes. Parenchymal transection was similar to OLR, except that vessels were ligated mainly by Hem-o-lock clips rather than sutures. The specimen was placed into a specimen bag and extracted out of the abdomen through a midline incision started from the periumbilical port side. A drainage tube was placed under the same circumstances as the OLR.

### Definitions

The type of liver resection was defined according to the Brisbane 2000 terminology [20]. Several studies had preliminarily but incompletely discuss the difficulty classification of LLR, mainly based on the extent and location of the resection. According to reports of Ban and Halls, [21, 22] which classified left lateral sectionectomy as low risk procedure and hepatectomy as moderate risk procedure, we defined single peripheral wedge resection and left lateral sectionectomy as *Extent I*, single segmentectomy (Sg 1, Sg 4a, Sg 4b and Sg 5–8), left lateral sectionectomy combined with single peripheral wedge resection and two peripheral wedge resections as *Extent II*; then, we took a single segmentectomy as the basic count factor and further defined bisegmentectomy, sectionectomy except left lateral sectionectomy and single segmentectomy combined with single peripheral wedge resection as *Extent III* (double risk factors); and major resection consisting of left hepatectomy, right hepatectomy and trisectionectomy as *Extent IV* (triple risk factors) [23]. In addition, we also redefined tumor location based on the Couinaud Segment Classification and surgical complexity [16, 24–26]. Sg 2 and Sg 3 were classified as *Location I*, as liver resection in this location was regarded as a procedure with relatively low complexity; the “*Difficult Segments*” Sg 1, Sg 7 and Sg 8 were classified as *Location III* for the relative high complexity of surgical procedure and the remaining Sg 4, Sg 5 and Sg 6 were classified as *Location II*. For patients with multiple lesions, location was determined by the largest lesion.

The definition of rPLC was described previously. The risk of patients receiving LLR was categorized into 3 levels, namely, low, moderate and high, according to the probability of undergoing risky surgery. To subjectively evaluate such risk, we used the operation time, the volume of total blood loss during surgery and postoperative hospital stays as reference parameters, for the operation time and blood loss indicating the difficulty of the surgical procedure, and the postoperative hospital days partly represented the major morbidity after surgery and the subsequent recovery. Thus, we defined risky surgery as the operation time, the total blood loss and postoperative hospital days all at or above the corresponding median value.

### Statistical analysis

Continuous variables were compared using two tail *t* test or Mann–Whitney test. Categorical variables were compared using χ^2^ test or Fisher’s exact test. Survival analysis was performed and compared using the Kaplan–Meier method and log-rank test. Propensity score matching (PSM) was executed to balance the baseline characteristics of the two groups using the MatchIt R package. The caliper score and match ratio were set at 0.01 and 1, respectively.

Patients in the LLR cohort were randomly assigned to the development cohort and internal validation cohort at a ratio of 2:1. Univariable logistic regression was conducted first in the development cohort, and then the variables with *P* values less than 0.1 as well as clinically relevant variables were included in the multivariable regression with a stepwise method to identify independent risk factors for risky surgery. The model was then presented graphically as a nomogram to predict risky surgery during LLR, the predictive accuracy of the model was measured by the concordance index (C-index) and calibration plots using the bootstrap resampling method. The decision curve and clinical impact curve were utilized for assessing the performance of the nomogram and addressing the cutoff values for the three risk levels according to the cost: benefit ratio [27]. The model was then applied to all patients. By comparing the intra- and postoperative outcomes of the high-, medium- and low-risk patients receiving LLR or OLR, we could determine the most appropriate resection approach for the certain risk patient.

A *P* value ≤ 0.05 was considered statistically significant, all analyses were carried out using SPSS (version 25.0: SPSS, Inc., Chicago, United States) or R (version 4.0.1: R Foundation, Vienna, Austria).

## Results

### Patients and survival

**Fig. 1:**
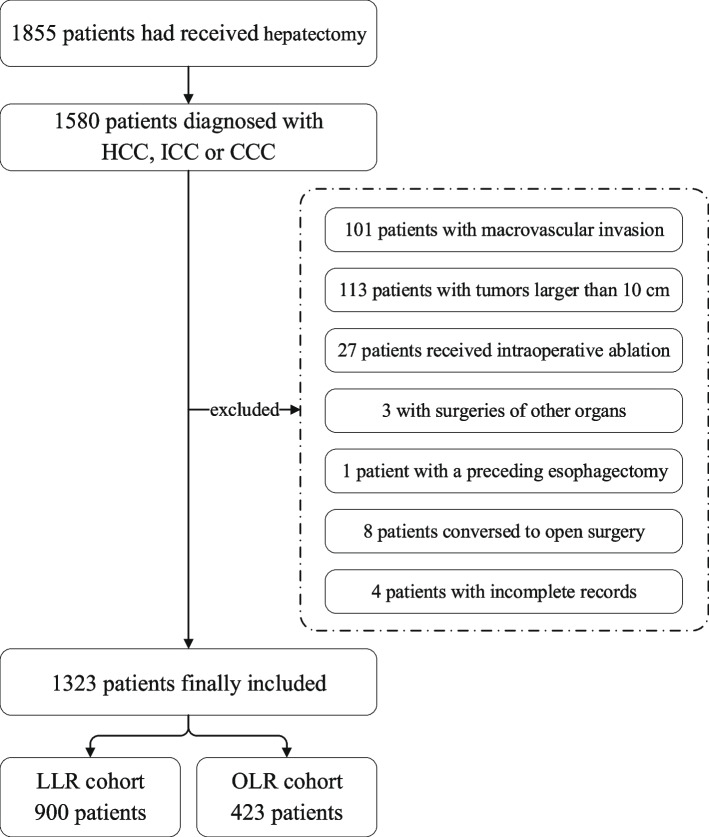
Patients Selection

A total of 1580 patients diagnosed with HCC had received hepatectomy from January 2013 to December 2018 in our center. Among them, 101 patients with macrovascular invasion and 113 patients with tumors larger than 10 cm were first excluded. Twenty-seven patients underwent intraoperative liver ablation, and three underwent surgeries of other organs simultaneously and were thus also excluded. One patient had undergone a preceding esophagectomy due to esophageal cancer, and eight patients who planned to receive LLR had conversion to OLR during the surgical procedure and were excluded. After removal of four patients due to incomplete records, 900 patients in the LLR cohort and 423 patients in the OLR cohort were finally included in the study (Fig. 1).

The baseline characteristics of the two groups of patients are listed in Table 1. Generally, the two groups of patients shared similar demographic and epidemiological characteristics. However, patients in the OLR group had larger tumor sizes, and the majority of tumors located in Sg8, while most tumors were located in the left lateral lobe in the LLR group. LLR had a similar median surgery time to that of OLR, but the mean blood loss was much lower. More than half of the patients in the LLR group underwent local wedge resection or left lateral sectionectomy (*Extent I*), while more patients in the OLR group underwent complicated major liver resection. After surgery, the median hospital stay was 5 days in the LLR group, which was significantly shorter than that in the OLR group. We first investigated whether LLR would bring out similar oncological benefit compared to OLR. Tumor diameter, tumor number, Child Pugh score, AFP level, the pre-defined tumor location and the pre-defined extent of resection were included for the PSM algorithm, since these variables were the most significantly unbalanced between the two groups and would potentially affect the decision of surgical approaches. After PSM, no difference was observed regarding to recurrence free survival (RFS) rate and overall survival (OS) rate between patients receiving LLR or OLR (*P* = 0.124 and 0.07, respectively) (Table S1 and Figure S1).

**Table 1:** Baseline Characteristics and Intra- and Postoperative Outcomes

	LLR cohort (*n* = 900)	OLR cohort (*n* = 423)	*P* value
Diagnosis			0.37
HCC	840 (93.3%)	389 (92.0%)	
ICC or CCC	60 (6.7%)	34 (8.0%)	
Age (Years)	55.0 (38.0–72.0)	54.0 (36.0–72.0)	0.53
Sex (Male; Female)	760; 140	360; 63	0.76
History of pre-treatment (Yes; No)	231; 669	123; 300	0.19
HBV infection (Yes; No)	765; 135	354; 69	0.54
Child Pugh score (5; 6)	857; 43	389; 34	0.02
ALBI grade (I; II)	843; 57	384; 39	0.07
Albumin (g/dl)	44.1 (40.3–47.9)	43.8 (39.8–47.8)	0.03
Total bilirubin (umol/L)	12.4 (6.3–18.5)	12.3 (6.7–17.9)	0.40
AFP (ng/ml)	32.3, 405.2	30.2, 810.7	0.06
Tumor diameter (cm)	3.5 (1.0–6.0)	6.0 (2.0–10.0)	< 0.001
Tumor number (Single; multiple)	729; 171	287; 136	< 0.001
Tumor location			< 0.001
Sg 1	13 (1.4%)	3 (0.7%)	
Sg 2/3	340 (37.8%)	51 (12.1%)	
Sg 4	102 (11.3%)	40 (9.5%)	
Sg 5	119 (13.2%)	42 (9.9%)	
Sg 6	194 (21.6%)	69 (16.3%)	
Sg 7	65 (7.2%)	78 (18.4%)	
Sg 8	67 (7.4%)	140 (33.1%)	
Tumor location (pre-defined)			< 0.001
*Location I*	340 (37.8%)	51 (12.1%)	
*Location II*	415 (46.1%)	151 (35.7%)	
*Location III*	145 (16.1%)	221 (52.2%)	
Extent of resection			
Wedge resection 1	314 (34.9%)	147 (34.8%)	< 0.001
Left lateral sectionectomy 2	168 (18.7%)	18 (4.3%)	
Single segmentectomy 3	116 (12.9%)	47 (11.1%)	
Bisegmentectomy 4	84 (9.3%)	68 (16.1%)	
Sectionectomy (except left lateral)	42 (4.7%)	30 (7.1%)	
Trisectionectomy 6	18 (2.0%)	28 (6.6%)	
Left hepatectomy 7	70 (7.8%)	27 (6.4%)	
Right hepatectomy 8	33 (3.7%)	17 (4.0%)	
Combined resection	55 (6.1%)	41 (9.7%)	
Extent of resection (pre-defined)			< 0.001
*Extent I*	482 (53.6%)	165 (39.0%)	
*Extent II*	155 (17.2%)	77 (18.2%)	
*Extent III*	142 (15.8%)	109 (25.8%)	
*Extent IV*	121 (13.4%)	72 (17.0%)	
Surgery time (minutes)	136.0 (60.0–212.0)	140.0 (105.0–175.0)	0.24
Pringle maneuver (Yes; No)	282; 618	263; 160	< 0.001
Hilar clamping duration (minutes)	25.0 (8.9–41.1)	15.0 (4.0–26.0)	< 0.001
Blood loss (mL)^*^	253.7, 9.0	333.6, 14.0	< 0.001
Post-operation hospital stays (days)	5.0 (2.0–8.0)	7.0 (5.0–9.0)	< 0.001
Surgical resection margin (cm)	1.5 (0.1–3.0)	1.0 (0.1–2.0)	< 0.001
Post-operation 180 days mortality	1.1% (10/900)	1.7% (7/423)	0.41

Abbreviations: *LLR* Laparoscopic liver resection,*OLR* Open liver resection, *HCC* Hepatocellular carcinoma, *ICC* Intrahepatic cholangiocarcinoma, *CCC* combined HCC and *ICC* History of pre-treatment, a history of previous antitumor treatment; HBV, Hepatitis B Virus; *AFP* Alpha-fetoprotein, *Sg* Couinaud segment

^*^ Blood loss is presented as the mean and standard error, other continuous variables are presented as the median and interquartile range

### Development and validation of the nomogram for predicting risky surgery

We then established a nomogram to predict risk surgery during LLR. Patients in the LLR group were randomly assigned to the training cohort and validation cohort at a ratio of 2:1. Risky surgery was confirmed in 214 patients in the training cohort. Univariate and multivariate logistic regression were conducted (Table 2). With results reported as odds ratio (95% CI), a history of previous antitumor treatment (1.66 [1.01–2.72]), tumor diameter (1.46 [1.29–1.66]), tumor location (3.70 [2.22–6.19] for *Location II* and 5.09 [2.67–9.70] for *Location III*) and resection extent (3.78 [2.19–6.52] for *Extent II*, 5.24 [2.87–9.56] for *Extent III* and 3.95 [2.05–7.61] for *Extent IV*) were independently associated with risky surgery.

**Table 2:** Univariable and Multivariable Analyses to Identify Predictors for Risky Surgery in LLR Based on Preoperative Data in the Development Cohort

	Univariate Logistic Regression		Multivariate Logistic Regression	
Variables	Odds Ratio (95% CI)	*P* value	Odds Ratio (95% CI)	*P* value
Diagnosis (HCC)	0.57 (0.34–0.97)	0.04		
Age (> 60 years)	1.07 (0.75–1.52)	0.73		
Sex (male)	0.96 (0.60–1.55)	0.87		
History of pre-treatment	0.93 (0.63–1.36)	0.71	1.66 (1.01–2.72)	0.05
HBV infection	0.70 (0.44–1.12)	0.14		
Child Pugh score (6)	0.56 (0.22–1.41)	0.22		
ALBI grade (II)	0.90 (0.44–1.83)	0.76		
Albumin	1.00 (0.94–1.05)	0.87		
Total bilirubin	0.98 (0.95–1.01)	0.25		
AFP (> 200 ng/ml)	1.20 (0.94–1.71)	0.31		
Resection margin	1.14 (0.81–1.60)	0.45		
Tumor diameter	1.61 (1.45–1.78)	< 0.001	1.46 (1.29–1.66)	< 0.001
Tumor number (multiple)	1.69 (1.11–2.57)	0.02		
Tumor location				
*Location II*	5.66 (3.65–8.78)	< 0.001	3.70 (2.22–6.19)	< 0.001
*Location III*	8.43 (4.86–14.60)	< 0.001	5.09 (2.67–9.70)	< 0.001
Extent of resection^#^				
*Extent II*	6.72 (4.08–11.08)	< 0.001	3.78 (2.19–6.52)	< 0.001
*Extent III*	13.91 (8.09–23.94)	< 0.001	5.24 (2.87–9.56)	< 0.001
*Extent IV*	10.35 (5.98–17.90)	< 0.001	3.95 (2.05–7.61)	< 0.001

Abbreviations: *HCC* Hepatocellular carcinoma, History of pre-treatment, a history of previous antitumor treatment, *HBV* Hepatitis B Virus, *AFP* Alpha-fetoprotein

^#^ As the extent of liver resection is usually planned ahead of surgery, this factor is considered as preoperative parament

**Fig. 2:**
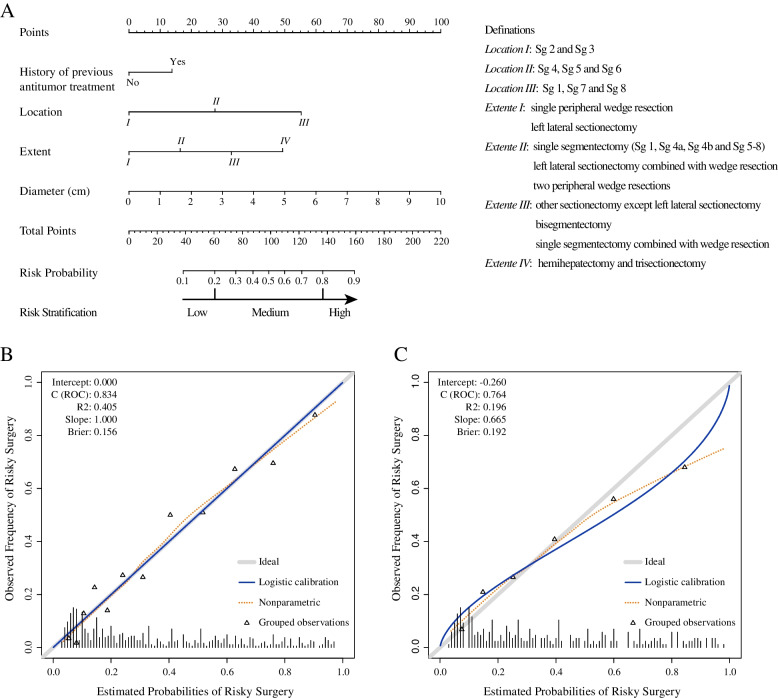
Nomogram (**A**) for preoperative prediction of risky surgery during laparoscopic liver resection and its calibration plots in (**B**) the development cohort and (**C**) validation cohort

**Fig. 3:**
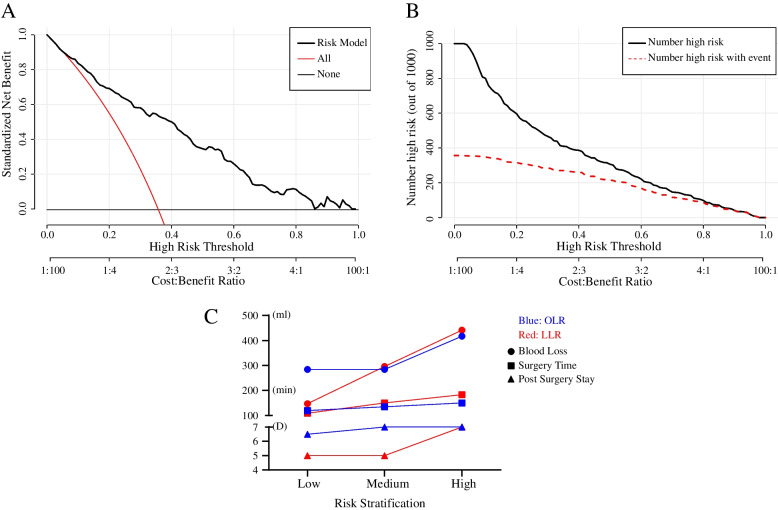
The decision curve (**A**) of the nomogram demonstrated the prediction model had the highest benefit across a wide range of values, together with the clinical impact curve. **B** Cutoff values of 0.2 and 0.8 were set to define the low-, medium- and high-risk for receiving LLR. **C** Differences of intra- and postoperative outcomes between corresponding risk levels patients receiving LLR or OLR indicated OLR was more dependable for high-risk patients, while LLR was better in low-risk patients. Abbreviations: LLR, laparoscopic liver resection; OLR, open liver resection; min, minute

These risk factors were used to establish a risky surgery nomogram (Fig. 2A). The nomogram illustrated tumor location as sharing the largest contribution to risky surgery during LLR. Each category or number of these risk factors was assigned a score on the point scale. By summing up the scores of each variable, the total score would indicate the probability of risky surgery through the scale beneath. For example, a treatment-naïve patients with an eight-centimeter tumor located in Sg 7/8 planned to receive right hepatectomy, the total points of this patient would be 187 and the corresponding probability of risky surgery during LLR exceeded 0.9. The nomogram demonstrated good accuracy in predicting risky surgery with a C index of 0.83 (95% CI 0.80–0.87). The calibration plot showed good agreement between the predicted risk of risky surgery and the observed risky surgery incidence (Fig. 2B). In addition, the decision curve of the risk model was ideally between the “invention for all” curve and the “invention for none” curve, which meant that the prediction model had the highest benefit across a wide range of values of preference (Fig. 3A). The calculated C index of the validation cohort was 0.76 (95% CI 0.71–0.82), with a good calibration curve for the risky surgery estimation (Fig. 2C).

### Clinical decision based on the nomogram

As the decision curve (Fig. 3A) and the clinical impact curve (Fig. 3B) showed, the cost/benefit ratio increased as the high-risk threshold increased. When the threshold was set to 0.8, the estimated number of risky surgery patients was nearly the same as the number of observed events, with a cost/benefit ratio for performing LLR of 4:1 and a specificity of 96.7% for predicting risky surgery among all patients in the LLR group using this nomogram. The cost/benefit ratio and the sensitivity were 1:4 and 88.2%, respectively, once the cutoff point was set to 0.2.

Hence, patients were stratified into high-, medium- or low-risk levels for receiving LLR if the calculated risk probability was more than 0.8, between 0.2 and 0.8 or less than 0.2, respectively. Then, all the included patients in our study were classified using this strategy. High-risk patients who actually underwent LLR had more blood loss (441 ml to 417 ml) and a longer surgery time (183 min to 150 min) than those who received OLR. While LLR demonstrated superiority in terms of blood loss, surgery time and postoperative hospital stay in low-risk patients (shown in Table 3). Moreover, receiving OLR slightly increased the surgery time and blood loss for high-risk patients compared with medium-risk patients, but the discrepancy was more significant in LLR, as the blood loss increased by 145 ml and the surgery time was prolonged by 33 min (Fig. 3C and Table 3).

**Table 3:** Intra- and Postoperative Outcomes of Corresponding Risk Levels Patients Receiving LLR or OLR

	Low Risk			Medium Risk			High Risk		
	LLR	OLR	*P* value	LLR	OLR	*P* value	LLR	OLR	*P* value
Surgery Time (min)	109.0 (56.0)	120.0 (50.0)	0.23	150.0 (55.1)	135.0 (30.0)	< 0.001	183.0 (73.0)	150.0 (38.0)	< 0.001
Blood Loss (ml)	147.2 (9.7)	284.1 (87.0)	0.002	295.9 (12.7)	283.8 (15.0)	0.55	441.1 (38.1)	417.1 (25.9)	0.59
Post-Surgery Stay (D)	5.0 (2.0)	6.5 (2.0)	0.001	5.0 (2.0)	7.0 (2.0)	< 0.001	7.0 (1.0)	7.0 (2.0)	0.10

Abbreviations: *LLR* Laparoscopic liver resection, *OLR* Open liver resection, *min* minute, *Post-Surgery Stay* Post-surgery hospital stays, *D* Day

We further analyzed the eight excluded LLR patients due to the conversion. Three of eight were identified as high-risk patients by our algorithm (risk probability 0.90, 0.97 and 0.98) and two were medium risk (risk probability 0.69 and 0.78), indicating the clinical-guiding value of our model.

## Discussion

In this study, we retrospectively reviewed patients with rPLC receiving LLR in our center and further divided LLR patients into high-, medium- and low-risk levels according to the probability of suffering risky surgery, which was assessed by the surgery time, total blood loss during the surgery and postoperative hospital stay. Using a multivariable logistic regression model, we confirmed a history of previous antitumor treatment, tumor diameter, tumor location and resection extent as risk factors for risky surgery and developed a nomogram to predict risky surgery during LLR. As far as we know, we presented the first report regarding intra- and post-operative outcomes of a certain group of patients receiving LLR and OLR. We found that high-risk patients would need less surgery time and have less blood loss during the operation if they had undergone OLR than if they had undergone LLR, with no difference in postoperative hospital stay. However, for low-risk patients, LLR may provide more safety both intraoperatively and postoperatively than OLR. What’s more, performing LLR in high-risk patients significantly increased surgical uncertainty and postsurgical complications compared with LLR in low-risk patients, but it seemed more stable and controllable to perform OLR at various risk levels. Hence, we recommended that patients classified into the high-risk level for LLR instead undergo OLR to reduce surgical risks and complications and that patients classified into the low-risk level undergo LLR to maximize the advantages of minimally invasive surgery. This nomogram using preoperative data to guide clinical decision is of great convenience and significance.

Laparoscopic surgery has long been used in cancer treatment and indeed holds several advantages, such as less postoperative pain, early intestinal motility and early ambulation but is not perfect [7]. Generally, surgeons tend to conduct LLR in patients with smaller and peripheral tumors. In our study, the majority of tumors were located in *Location II* (Sg 4, Sg 5 and Sg 6) in the LLR group and in *Location III* (Sg 1, Sg 7, Sg 8) in the OLR group. LLR group patients mainly underwent wedge resection and left lateral sectionectomy, while more bisegmentectomies and hemihepatectomies were conducted in the OLR group. This finding is consistent with Cheung's and Witowski's reports and may partly explain why OLR requires more surgery time and has higher total blood loss [9, 10]. We were concerned that the superiority of LLR in the dominant aspect (such as for left lateral sectionectomy) would conceal its weakness in complex sections (such as the *Difficult Segments* and major resection) [16, 28, 29]. Therefore, we developed a nomogram to nail down certain subgroups of patients for whom OLR would be more appropriate than LLR.

We found that a history of previous antitumor treatment was associated with a high probability of undergoing risky surgery during LLR. As HCC is a frequently recurrent cancer involving multidisciplinary treatments due to its etiological and biological nature, [30] many patients have received various therapies, such as first surgery, radiofrequency ablation (RFA) and transarterial chemoembolization (TACE), before preparing for another surgery. Intra-abdominal adhesion was inevitable in these patients, and the reported adhesion rate was up to 64% for previous LR, 57% for RFA and 89% for TACE [31–34]. Performing LLR for those patients required a longer operative time and was associated with a greater frequency of intraoperative complications, such as liver laceration, bile leakage and diaphragmatic tears, [33, 34] which increased the surgery time and postsurgical hospital stay, resulting in unfavorable short-term outcomes for patients. With a wider surgical vision field and anatomical structure exposure, OLR might be a safer approach for treated or recurrent patients.

In previous studies, Daisuke et al. included liver function, tumor size, tumor location, resection extent and proximity to Glisson’s tress to generate a difficulty index for LLR. Halls and his colleagues included lesion size, lesion type, classification of resection, open liver resection history and neoadjuvant chemotherapy to define difficult LLR, and Yoshikuni et al. simply classified LLR into three difficulty levels according to resection type [21, 22, 35]. Those studies focused on the laparoscopic operation itself of difficulty but lacked clinical practice guiding. As more and more complexity classifications were proposed, the clinical problem has gradually shifted from identifying difficult or risk surgeries to making it easier to identify risk surgeries and how to deal with risk surgeries. In this study, we integrated the previously reported risk factors for LLR, removed insignificant factors through the regression model of a large sample of patients, retained and redefined tumor location and resection extent as *Locations I*, *II*, and *III* and *Extents I*, *II*, *III*, and *IV* based on surgical difficulty according to clinical experience and other reports. Tumor location and resection extent were the two most powerful factors predicting risky surgery for LLR, especially with the combination of *Location III* and *Extent III* or *IV*, which is known to involve complex liver resection such as right posterior sectionectomy and right hepatectomy [36]. Our nomogram using preoperative data and objective outcome indicators was able to distinguish the three risk levels of patients receiving LLR before surgery with great practicality and convenience.

What’s more, we also revealed that high-risk patients benefited more from OLR than from LLR. In high-risk patients, OLR was able to reduce the surgery time and total blood loss but not prolong postoperative hospital stays compared to LLR. This finding was partially consistent with Yoon’s study comparing laparoscopic to open right hepatectomy in cirrhotic HCC patients, in which LLR remarkably increased the operative time, while the length of postoperative hospital stay was shorter and the difference in blood loss remained statistically nonsignificant [8]. We thought this conclusion was of great value for clinical practicing and patients caring. With the improvements in techniques and equipment, LLR is now widely performed for the treatment of rPLC and the acknowledged “difficult surgeries” such as laparoscopic right posterior sectionectomy and right hepatectomy are wildly performed by surgeons. However, the patient safety should always come to first when deciding the operation approaches. We compared the outcomes of patients receiving LLR and OLR head to head and revealed that OLR reduced the surgery time and total blood loss but not prolonged postoperative hospital stays in high-risk patients, which meant OLR might be more safer and proper for those patients and should be the first choice in most cases. In addition, the operation time and blood loss increased notably in high-risk patients compared to medium-risk patients in LLR, indicating the higher uncertainty and hazard for performing LLR in those patients. Hence, we recommend that only experienced surgeons who regularly perform LLRs for medium-risk patients were suitable for conducting LLR for those high-risk patients. In contrast, OLR was more stable and controllable within the three stratifications, although this kind of stability seemed less beneficial in low-risk patients.

The present study has several limitations. First, this was a single-center retrospective study in which selection bias was inevitable even with the large number of patients we included. In addition, patients in our study were all Child Pugh A class due to the strict surgical criteria in our center, and the impact of underlying liver function may be underestimated in our model. Nevertheless, our study represents a major part of HCC patients receiving hepatectomy. Patients are more homogeneous, and the model is well fitted in the development and validation cohorts. The reliability and validity of our model should be verified by other groups, even RCTs. Second, we evaluated postoperative complications by posthospital stay, from which some acute and subjective postsurgical responses, such as incision pain, were omitted. However, we focused mainly on the severe complications that need inpatient intervention and prolonged hospital stay to identify risky surgery, decreased incision pain after surgery, though a definite advantage of LLR, was less essential to our study object.

## Conclusions

In conclusion, we divided patients into high-, medium- and low-risk groups for undergoing LLR using a novel nomogram we proposed and further compared the outcomes of patients at the same risk level who underwent LLR or OLR head to head. We suggest that OLR is more dependable for high-risk patients, only experienced surgeons who regularly perform LLRs for medium-risk patients are suitable for conducting LLR for those high-risk patients. LLR is more suitable for low-risk patients regarding patients’ outcomes and surgical risks. Our results provide a new perspective to balance the choice between LLR and OLR for surgeons.

## Supplementary Information

**Additional file 1:** **Figure S1.** The recurrence free survival (A) and overall survival (B) of patients in the LLR and OLR group after PSM. Abbreviations: PSM, propensity score matching; LLR, laparoscopic liver resection; OLR, open liver resection; RFS, recurrence free survival; OS, overall survival.

**Additional file 2:** **Table S1.** Baseline Characteristics after PSM.

## Data Availability

The datasets used during the current study are available from the corresponding author on reasonable request.

## Abbreviations

HCC: Hepatocellular carcinoma
LR: Liver resection
OLR: Open liver resection
LLR: Laparoscopic liver resection
RCT: Randomized controlled trial
rPLC: Resectable primary liver cancer
