# Supplementary material for: An integrated strategy for deciding open versus laparoscopic hepatectomy for resectable primary liver cancer
Source: BMC Cancer. 2023 Feb 27;23:193. doi: 10.1186/s12885-023-10630-x (PMC9972775; doi:10.1186/s12885-023-10630-x)
Supplement: Supplementary file 1 — Additional file 1: Figure S1. The recurrence free survival (A) and overall survival (B) of patients in the LLR and OLR group after PSM. Abbreviations: PSM, propensity score matching; LLR, laparoscopic liver resection; OLR, open liver resection; RFS, recurrence free survival; OS, overall survival. [file 12885_2023_10630_MOESM1_ESM.pdf]

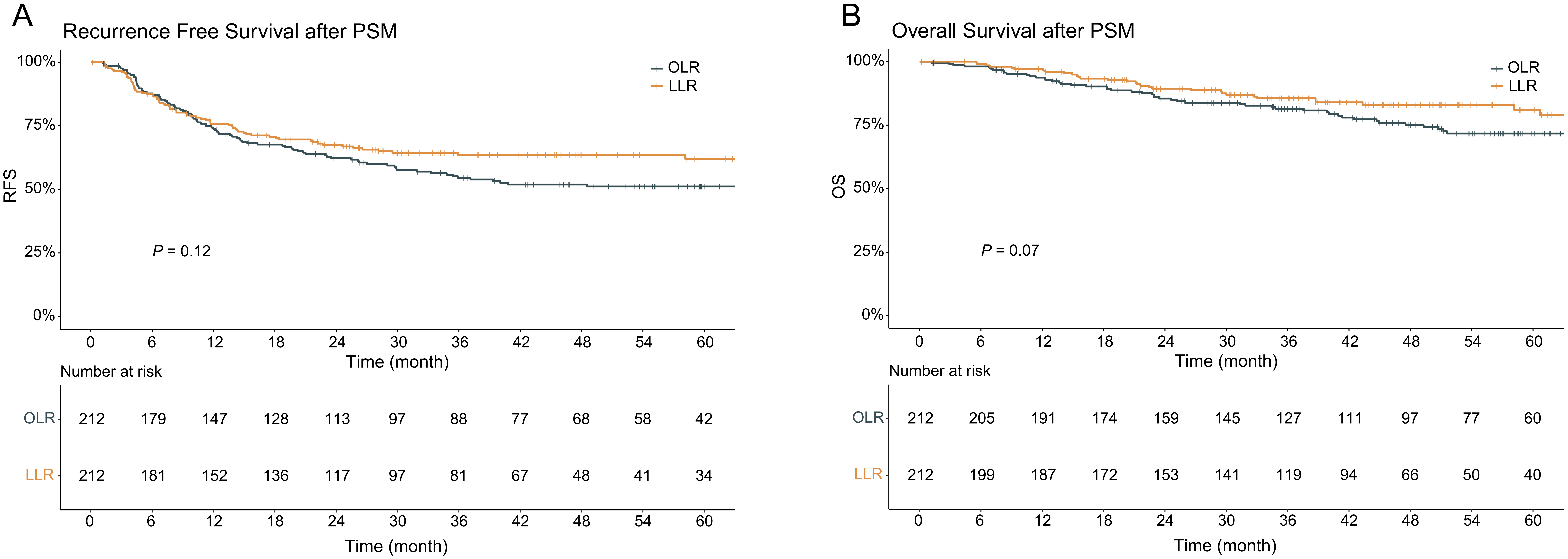

Figure S1. The recurrence free survival (A) and overall survival (B) of patients in the LLR and OLR group after PSM.
Abbreviations: PSM, propensity score matching; LLR, laparoscopic liver resection; OLR, open liver resection; RFS, recurrence free survival; OS, overall survival.
