# Supplementary material for: An integrated strategy for deciding open versus laparoscopic hepatectomy for resectable primary liver cancer
Source: BMC Cancer. 2023 Feb 27;23:193. doi: 10.1186/s12885-023-10630-x (PMC9972775; doi:10.1186/s12885-023-10630-x)
Supplement: Supplementary file 2 — Additional file 2: Table S1. Baseline Characteristics after PSM. [file 12885_2023_10630_MOESM2_ESM.docx]

Table S1. Baseline Characteristics after PSM

|  | LLR cohort (n=212) | OLR cohort (n=212) | *P* value |
| --- | --- | --- | --- |
| Diagnosis |  |  | 0.847 |
| HCC | 197 (92.9%) | 198 (93.4%) |  |
| ICC or CCC | 15 (7.1%) | 14 (6.6%) |  |
| Child Pugh score |  |  | 0.284 |
| 5 | 198 (93.4%) | 203 (95.8%) |  |
| 6 | 14 (6.6%) | 9 (4.2%) |  |
| AFP (ng/ml) | 13.3, 209.6 | 18.5, 309.9 | 0.218 |
| Tumor diameter (cm) | 5.0, 2.2 | 5.0, 2.3 | 0.906 |
| Tumor number |  |  | 0.492 |
| Single | 165 (77.8%) | 159 (75.0%) |  |
| Multiple | 47 (22.2%) | 53 (25.0%) |  |
| Tumor location (pre-defined) |  |  | 0.431 |
| *Location I* | 33 (15.6%) | 39 (18.4%) |  |
| *Location II* | 103 (48.6%) | 90 (42.4%) |  |
| *Location III* | 76 (35.8%) | 83 (39.2%) |  |
| Extent of resection (pre-defined) |  |  | 0.784 |
| *Extent I* | 91 (42.9%) | 82 (38.7%) |  |
| *Extent II* | 34 (16.0%) | 40 (18.9%) |  |
| *Extent III* | 50 (23.6%) | 50 (23.5%) |  |
| *Extent IV* | 37 (17.5) | 40 (18.9%) |  |

Abbreviations: PSM, propensity score matching; LLR, laparoscopic liver resection; OLR, open liver resection; HCC, hepatocellular carcinoma; ICC, intrahepatic cholangiocarcinoma; CCC, combined HCC and ICC; AFP, alpha-fetoprotein.
